# Investigation of *Linum flavum* (L.) Hairy Root Cultures for the Production of Anticancer Aryltetralin Lignans

**DOI:** 10.3390/ijms19040990

**Published:** 2018-03-26

**Authors:** Sullivan Renouard, Cyrielle Corbin, Samantha Drouet, Barbara Medvedec, Joël Doussot, Cyril Colas, Benoit Maunit, Avninder S. Bhambra, Eric Gontier, Nathalie Jullian, François Mesnard, Michèle Boitel, Bilal Haider Abbasi, Randolph R. J. Arroo, Eric Lainé, Christophe Hano

**Affiliations:** 1 Laboratoire de Biologie des Ligneux et des Grandes Cultures, LBLGC EA1207/INRA USC1328, Team Plant Lignans, Université d’Orléans, 28000 Chartres, France; sullivan.renouard@univ-orleans.fr (S.R.); cyrielle.corbin@outlook.com (C.C.); samantha.drouet@etu.univ-orleans.fr (S.D.); barbara.medvedec@gmail.com (B.M.); joel.doussot@cnam.fr (J.D.); eric.laine@univ-orleans.fr (E.L.); 2 Ecole Sciences Industrielles et Technologies de l’Information, SITI Département CASER, Le CNAM, 75141 Paris, France; 3 Institut de Chimie Organique et Analytique, ICOA UMR7311, Université d’Orléans-CNRS, 45067 Orléans, France; cyril.colas@univ-orleans.fr (C.C.); benoit.maunit@univ-orleans.fr (B.M.); 4 Faculty of Health and Life Sciences, De Montfort University, The Gateway, Leicester LE1 9BH, UK; abhambra@dmu.ac.uk (A.S.B.); rrjarroo@dmu.ac.uk (R.R.J.A.); 5 Biologie des Plantes et Interactions, BIOPI EA3900, Biologie des Plantes et Innovation, Université de Picardie Jules Verne, F-80037 Amiens, France; eric.gontier@u-picardie.fr (E.G.); nathalie.jullian@u-picardie.fr (N.J.); francois.mesnard@u-picardie.fr (F.M.); michele.boitel@u-picardie.fr (M.B.); 6 Department of Biotechnology, Quaid-i-Azam University, Islamabad 45320, Pakistan; bhabbasi@qau.edu.pk

**Keywords:** aryltetralin lignans, deoxypodophyllotoxin, 6-Methoxy-podophyllotoxin, podophyllotoxin, hairy root, *Linum flavum*

## Abstract

*Linum flavum* hairy root lines were established from hypocotyl pieces using *Agrobacterium rhizogenes* strains LBA 9402 and ATCC 15834. Both strains were effective for transformation but induction of hairy root phenotype was more stable with strain ATCC 15834. Whereas similar accumulation patterns were observed in podophyllotoxin-related compounds (6-methoxy-podophyllotoxin, podophyllotoxin and deoxypodophyllotoxin), significant quantitative variations were noted between root lines. The influence of culture medium and various treatments (hormone, elicitation and precursor feeding) were evaluated. The highest accumulation was obtained in Gamborg B5 medium. Treatment with methyl jasmonate, and feeding using ferulic acid increased the accumulation of aryltetralin lignans. These results point to the use of hairy root culture lines of *Linum flavum* as potential sources for these valuable metabolites as an alternative, or as a complement to *Podophyllum* collected from wild stands.

## 1. Introduction

**Figure 1:**
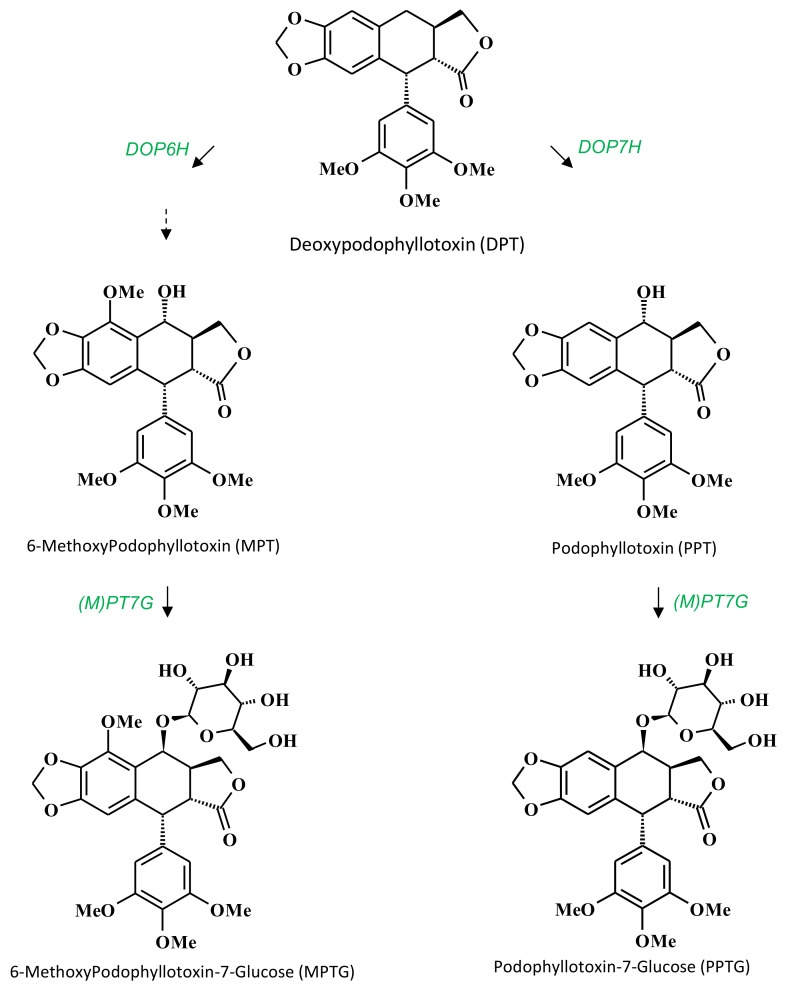
Structures of the main ATL (aryltetralin lignan) and the corresponding putative biosynthetic pathway leading from DPT to PPTG and MPTG in *L. flavum*. DOP6H: DPT-6-hydroxylase, DOP7H: DPT-7-hydroxylase, (M)PT7G: MPT- and/or PPT-7-glucosyltransferase, DPT: deoxypodophyllotoxin, MPT: methoxypodophyllotoxin, MPTG: methoxypodophyllotoxin-7-glucoside, PPT: podophyllotoxin, PPTG: podophyllotoxin-7-glucoside.

Podophyllotoxin (PPT; Figure 1) is a well-known aryltetralin lignan (ATL), which serves as a unique starting compound for the semi-synthesis of leading anticancer drugs [1]. These drugs, including etoposide, teniposide and etopophos, are known to inhibit topoisomerase II and are widely used to treat several types of cancers [2,3]. PPT is also known to inhibit the *Herpes simplex* type I virus replication [4] and some of its semi-synthetic derivatives possess pronounced anti-HIV properties [5]. PPT and other related ATLs are extracted from *Podophyllum hexandrum*, a perennial herb found in the Himalayas which accumulates high amounts of this compound, around 4% of DW (Dry Weight) in its roots and rhizomes [6]. However, due to over-collection and a lack of cultivation, this species is now endangered [1]. Total synthesis of podophyllotoxin is currently not a commercially viable solution due to its specific stereochemical structure with four chiral centers. Therefore, the PPT-supply issue triggered an active search for alternative natural sources [7].

Several alternative sources of PPT have been identified during the last decades including Cupressaceae, Lamiaceae, Linaceae, Podophyllaceae and Polygalaceae (see [8] for review). Considering their lignan accumulation capacities, some *Linum* species are considered as a promising alternative source of PPT [9,10,11,12]. The genus *Linum* (Linaceae) comprises more than 230 species, largely distributed among temperate and subtropical climates [11] and *Linum* species from the section *Sylinum*, including *Linum flavum*, have been reported to accumulate high amount of ATL in their aerial parts, roots and seeds [13,14,15]. *L. flavum* seeds constitute the richest *Linum* source of ATL [15]. However, contrary to its congener, the common flax *L. usitatissimum*, *L. flavum* (aka golden flax or yellow flax) is a non-cultivated small-erected woody perennial plant, native from Central Europe, with a poor agricultural potential (e.g., slow growth or seed dehiscence). Therefore, in vitro plant cell and/or tissue cultures constitute an attractive alternative to whole *L. flavum* plant for the production of these valuable metabolites.

Cell suspension or callus cultures of different *Linum* species, such as *L. album*, *L. flavum*, *L. mucronatum* or *L. nodiflorum,* have been initiated and shown to produce ATL [16,17,18,19,20,21]. The cell line selection and use of optimized culture conditions resulted in accumulation levels of 1.7% DW of 6-methoxypodophyllotoxin (MPT, Figure 1) in *L. nodiflorum* cell suspension [22] and 0.8% DW of PPT in *L. album* cell suspension [23]. Elicitation of *L. nodiflorum* cell suspension with indanoyl-isoleucine resulted in the highest ALT yield obtained for this in vitro culture system using a *Linum* species: i.e., 2.5% DW accumulation of MPT [24]. However, comparatively, hairy root (HR) cultures of *L. flavum*, *L. album*, *L. persicum*, *L. strictum* and *L. mucronatum* produced about twice as much ATL as cell suspensions [23,25,26,27,28]. HR lines have been described for their comparable or even greater production of secondary metabolite because of higher differentiation compared to plant cell tissue and organ culture systems such as cell suspensions [29]. *Agrobacterium rhizogenes* is a Gram negative soil bacterium able to induce hairy roots (HR) phenotype following genes transfer to the wounded plants [30]. HR phenotype is characterized by vigorous growth, lack of geotropism, high lateral branching and genetic stability in hormone free medium [30]. *L. flavum* HR lines have been previously established and described as an effective system for the production of ATL including up to 3.5% DW of MPT [25,31] or lignan precursor such as coniferin [32]. However, in these studies few independent HR lines have been generated and analyzed while in the absence of a thorough assessment of the influence of growing conditions on ATL accumulation it is difficult to get a clear idea of the production potential of this species.

In the present work, we report on the establishment of fourteen HR lines as well as their physiological, molecular and biochemical characterizations leading to the selection of a productive *L. flavum* HR line. Then, a complete scale of experiments, including the selection of adequate culture medium, carbon sources as well as the use of several elicitors, permeation and precursor feeding, were next evaluated to further optimize ATL accumulation and investigate the potential of *L. flavum* HR as a production system of anticancer ATL.

## 2. Results and Discussion

### 2.1. HR Induction and Molecular Characterization

**Figure 2:**
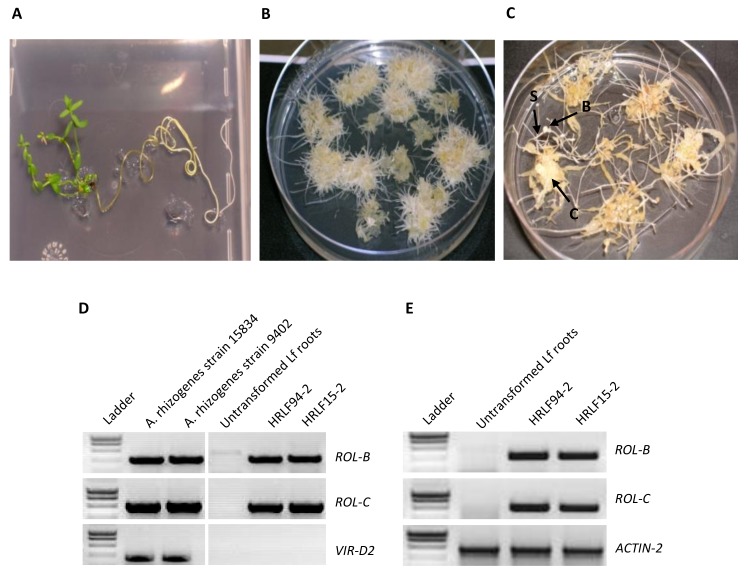
*A. rhizogenes*-mediated transformation and molecular characterization of the resulting *L. flavum* HR lines. (**A**) Germination of wild type *Linum flavum*; (**B**) Hairy roots of *Linum flavum*, line HRLF15.2; (**C**) Hairy roots of *Linum flavum*, line HRLF94-6 (C: callus, B: bud, S: shoot); (**D**) PCR amplified DNA fragments of *ROL-B*, *ROL-C* and *VIR-D2* pro-oncogenes from the plasmidic DNA of *A. rhizogenes* strains or genomic DNA of *L. flavum*. From left to right: Ladder, DNA from the plasmids of *A. rhizogenes* LBA 9402 and ATCC 15834 strains respectively, used as positive control, genomic DNA of wild type roots *L. flavum* cultivated in vitro used as negative control, genomic DNA from two transgenic *L. flavum* hairy roots lines, HRLF94-2 and HRLF15-2 respectively. (**E**) sqRT-PCR analysis of *ROL-B* and *ROL-C* pro-oncogene expressions in *L. flavum* wild type roots and in two transgenic *L. flavum* hairy roots lines, HRLF94-2 and HRLF15-2 respectively. Total RNAs isolated from hairy roots were subjected to sqRT-PCR analysis using *ACTIN-2* gene as internal control. Ten microliters of RT-PCR products were loaded on 1% (*w*/*v*) agarose gel.

The leaf disc technique was previously shown as preferable for hairy root induction in *L. flavum* [32] and was therefore employed and compared with induction from hypocotyls. In our hands, a marked difference was observed since no hairy root induction was observed using cotyledon disks as starting explants whereas a good transformation rate was obtained using hypocotyls pieces. The influence of the Agrobacterial strain used for the induction was also evaluated using two different strains: ATCC 15834 and LBA 9402. After 4 weeks of cultivation individual lines were isolated from different individual explants, denoted as HRLF15.1 to HRLF15.18, for lines initiated by *A. rhizogenes* strain ATCC 15834, and HRLF94.1 to HRLF94.16, for that initiated by the LBA 9402 strain. They were next cultivated on hormone free medium containing antibiotics in order to eliminate the remaining bacteria. No clear differences were observed in the ability of the two strains to induce transformation on *L. flavum* explants (16% of induction for ATCC 15834 versus 18% of induction for LBA 9402). The induction rate obtained with the ATCC 15834 strain was higher to that observed by Lin et al. [32] with this strain. The induction rate obtained with the LBA 9402 strain was comparable to that observed by Oostdam et al. [25] but lower to that previously reported by Lin et al. [32]. Following eight additional weeks (i.e., four additional subcultures on hormone free medium containing antibiotics), individual HR lines were transferred on hormone free medium without antibiotics and used for analysis. The *L. flavum* HR lines produced by the ATCC 15834 strain exhibited a true hairy root phenotype (Figure 2B) associated with a vigorous growth (Figure S2). However, not all isolated HR lines of *L. flavum* displayed both a hairy root phenotype and a vigorous growth, in particular for the lines resulting from LBA 9402 transformation. Indeed, during this period, some of the hairy root lines have stopped their growth and eventually died. Other HRLF94 lines showed an interesting growth index but presented morphological instability, including the development of callus and/or shoots (Figure 2C). Contrary to Lin et al. [32], Oostdam et al. [25] also described such a phenomenon in their *L. flavum* hairy root lines initiated from this LBA 9402 strain of *A. rhizogenes*. Lin et al. [32] speculated that this observed morphological instability could be the consequence of the presence of zeatin in the culture medium used by these authors for hairy root induction. But we also observed these morphological changes in the absence of any growth regulator and zeatin in particular. The LBA 9402 strain belongs to the agropine-mannopine type whereas ATCC 15834 is of agropine type. The ability of mannopine type strain to induce somatic embryos in hairy roots has been observed in *Cucurbita pepo,* whereas agropine type strains failed to produce hairy roots [33].

Beside these morphological characterizations, the confirmation of the complete and stable transformation status of the isolated HR lines was completed by a molecular analysis. An amplification signal at 540 and 770 bp for ROL-B and ROL-C fragments, respectively, was observed in transgenic hairy roots (Figure 2D), which is a proof for the successful genetic transformation. No amplification signal was detected for VIR-D2 gene in the same lines, indicating the absence of residual bacteria. The Ri plasmid of *A. rhizogenes* ATCC 15834 and LBA 9402 were used as positive controls (Figure 2D) whereas untransformed roots were used as negative controls (no amplification signal) (Figure 2D). Moreover, the expression of the two pro-oncogenes *ROL-B* and *ROL-C* was confirmed in transgenic lines by sqRT-PCR (Figure 2E).

### 2.2. Screening and Selection of Efficient HR Genotype

Fourteen *L. flavum* hairy root lines were screened for their growth and ATL production and compared to in vitro untransformed *L. flavum* roots (Table 1). After six times subculturing (three months) on medium containing antibiotics, HR lines were transferred to antibiotic-free B5 medium and the growth indices were determined at day 20 of cultivation. The growth indices varied from 3.4 to 5.9 with higher values measured for the LBA 9402-derived HR lines (Table 1). The liquid chromatography (LC) and LC-High Resolution-Mass Spectrometry analysis indicated the same pattern of accumulation for each analyzed line but significant quantitative variations were observed. The two mains peaks corresponded to MPT and MPT-G as main ATL accumulated in *L. flavum* HR as already described by Oostdam et al. [25]. Except for some HR line including the HRLF94.4 and HRLF94.8, which accumulated low amount of ATL compared to other HR lines, MPT is mainly accumulated under its glucosylated form (i.e., MPT-G). HR lines accumulated up to 24.1 and 8.4 mg·g^−1^ of MPT-G and MPT respectively (for HRLF94-2). Oostdam et al. [25] have reported similar MPT contents (from 1.5% to 3.5% of total MPT) in their *L. flavum* HR lines. Wichers et al. [34] have compared the MPT contents of plant parts of *L. flavum* grown under different conditions (field-grown, greenhouse and in vitro agar-grown plants). The total MPT content (MPT + MPTG) of 0.4% DW reported by these authors [34] for in vitro agar-grown roots of *L. flavum* is good agreement with our result (in vitro Wild Type *L. flavum* root, Table 1). Interestingly, these authors reported that the MPT content was 10 times higher in the roots of in vitro agar-grown plants than in the roots of soil-grown plants [34]. This observation point the great potential of *L. flavum* HR as a production source of ATL. Here, significant amounts of other AT lignans such as PPT-G and PPT as well as DPT were also detected in all tested HR lines. These results showed it was possible to select HR lines with vigorous growth and high ATL production. Based on these data (phenotype stability, growth and lignan accumulation) the *L. flavum* line HRLF15.2 was chosen as elite line for further optimization and analysis for ATL production.

Other *Linum* species have led to HR lines producing ATL. Under non-optimized conditions, a single HR line of *L. strictum* produced only 0.57 mg·g^−1^ DW MPT [28] whereas several studies have shown that the used of selected HR lines and/or optimized culture conditions could result in significant increases of the ATL production yields: 37–48 mg·g^−1^ MPT in *L. album*, 40–56 mg·g^−1^ MPT in *L. persicum* [23], 4.55 mg·g^−1^ DW PPT and 41.38 mg·g^−1^ DW MPT in *L. mucronatum* [26]. Our next objectives were to evaluate the impact of different culture conditions to improve the ATL accumulation in *L. flavum* HR.

### 2.3. Improvement of Culture Conditions

**Figure 3:**
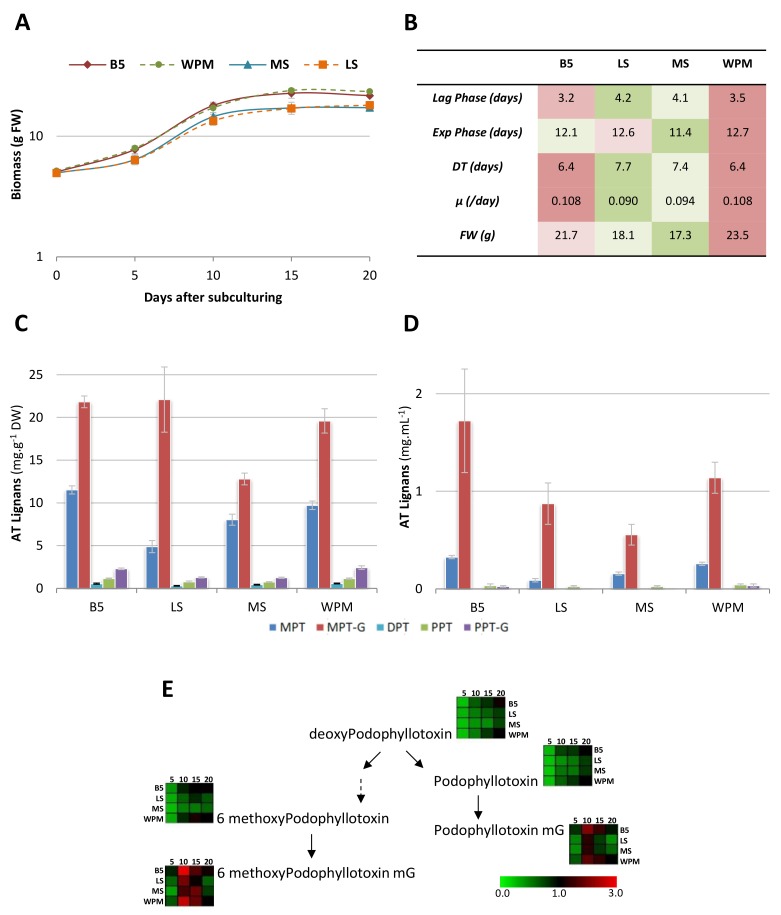
Impact of basal medium composition on the growth characteristics and ATL accumulation of the HRLF15.2 line. (**A**) Growth kinetics of *Linum flavum* HR (in g of fresh weight) cultivated in flasks containing 100 mL of B5, WPM, MS and LS culture media; (**B**) Growth characteristics of the HRLF15.2 line in the B5, WPM, MS and LS culture media; (**C**) Intracellular accumulation of the main ATL and their glucosylated forms in HRLF15.2 line growing in the B5, WPM, MS and LS culture media; (**D**) Extracellular accumulation of the main ATL and their glucosylated forms in HRLF15.2 line growing in the B5, WPM, MS and LS culture media; (**E**) Relative intracellular accumulation kinetic of the main ATL and their glucosylated forms in HRLF15.2 line growing in the B5, WPM, MS and LS culture media. Detailed ATL accumulation kinetics are presented in Figure S1. Each point is the mean and standard deviation of the three independent experiments.

**Figure 4:**
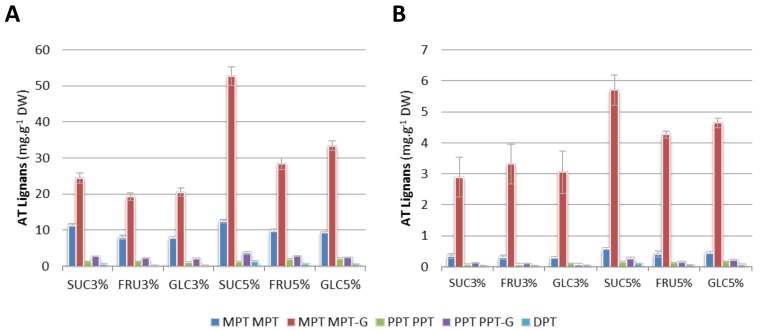
Influence of carbon source the ATL accumulation of the HRLF15.2 line. (**A**) Intracellular accumulation of the main ATL and their glucosylated forms in HRLF15.2 line growing in the B5 medium supplemented with 3% or 6% (*w*/*v*) sucrose (SUC), fructose (FRU) or glucose (GLC); (**B**) Extracellular accumulation of the main ATL and their glucosylated forms in HRLF15.2 line growing in the B5 medium supplemented with 3% or 6% (*w*/*v*) sucrose (SUC), fructose (FRU) or glucose (GLC). Growth kinetic are presented in Figure S2. Each point is the mean and standard deviation of the three independent experiments.

The composition of plant culture media is an important parameter for both growth and specialized metabolite production [35,36]. Here, the effects of mineral composition on the growth and AT accumulations were evaluated using four different culture media: Gamborg B5 (B5 [37]), Linsmaier and Skoog (LS [38]), Murashige and Skoog (MS [39]) and Woody Plant Medium (WPM [40]). The ion contents of these basal media are presented in supplementary data (Table S3). The main differences between the mineral bases of these media are the amount and form of the nitrogen and the abundance in anions, MS and LS being high in total nitrogen and WPM high in sulfate. LS and MS medium only differed by their vitamin composition. The HRLF15-2 line was used as elite line and both its growth and ATL production over 20 days were recorded. Typical sigmoid growth curves were observed for HR lines cultivated in the four different media (Figure 3A). Determination of the lag, exponential and stationary phases observed for each media allowed us to calculate the doubling time (DT) as well as the specific growth rate per day (µ in day^−1^, Figure 3B) as previously described by Mairet et al. [41]. As indicated in Figure 4, the highest growth rates were observed with the B5 and WPM media having very close specific growth rate and dry weight production along the considered culture period. However, we noted higher degeneration and morphological changes in our HR lines using WPM medium after 15 days. HR line growing in LS and MS media showed a reduced growth in comparison to WPM and B5 media. Baldi et al. [42] also reported higher biomass for *Linum album* HR lines produced in B5 medium as compared to MS medium. Here no significant difference was observed for the growth of *L. flavum* HR lines in LS and MS media as previously reported by Lin et al. [32].

Whichever medium was used, MPT and its glycoside form MPTG appear to be the main ATL accumulated in HR and their culture medium, certainly because of root exudation. DPT, PPT and PPTG were also detected in both HR and culture medium (Figure 3C,D). Highest accumulation and exudation yields were measured using B5 medium. ATL accumulation kinetics revealed the same pattern for each culture media (Figure 3E and Figure S1) with a maximal accumulation between days 10 and 15 followed by a decline period up to day 20 peculiarly pronounced for the ATL glycosides MPTG and PPTG; the amounts of their aglycone forms being more stable during the same period. Interestingly, these observations are consistent with our previous work [31] in which we have observed, in normal growth conditions, a coordination of glycosylation steps leading to MPTG and PPTG perhaps through the action of the same enzyme or through the coordination of the gene expression regulation of two distinct enzymes which remain to be identified. Low nitrogen containing media (B5 and WPM) were found to be more efficient both for growth and development and for ATL production. Nitrogen (N) is one of the main nutrients required for plant growth and is therefore generally applied in significant amounts to ensure important yields. However, some recent studies have shown that low nitrogen could contribute to higher secondary metabolite production. Phenylalanine ammonia lyase (PAL) activity (a key enzyme of the phenylpropanoid pathway catalyzing the deamination of l-Phe to *trans*-cinnamic acid) and flavonoid accumulation were higher in *Labisia pumila* grown under low nitrogen fertilization [43]. Both saponin and flavonoid production were increased in in vitro cultures of *Clidemia hirta* growing in culture medium containing lower nitrogen content [36]. Adjusting media for in vitro plant culture generally focuses on growth regulators; our results evidenced that a particular attention should be paid to the basal culture medium for the production of secondary metabolites.

Altogether, these results pointed B5 medium and 14-day culture cycle as optimum for the growth and ATL production in our HRLF15.2 elite line. Here the use of optimal culture medium resulted in a 10% productivity gain with a total MPT content reaching 3.3% DW.

### 2.4. Impact of Carbon Source and Concentrations

As most in vitro cultures, HR are heterotrophically-grown culture; hence a carbon source has to be provided through their culture medium and simple sugars are basic carbon sources used to ensure plant growth and metabolite production. Both the type and the level of sugars has been shown to greatly affect the productivity of secondary metabolites accumulated in plant cell and tissue cultures [35]. Beside their simple nutrition effect, high sugar concentration could result in an osmotic stress that can affect plant secondary metabolism as described for sucrose [35]. Regarding the carbon source used for plant in vitro cultures producing lignans we can note that 3% sucrose was frequently employed [11]. Here three different sugars were tested as carbon source: glucose, fructose and sucrose at two different concentration levels: 3% and 6% (*w*/*v*). Best results were obtained using sucrose at 6% (*w*/*v*) concentration level (Figure 4A,B and Figure S2) a with total MPT content of 6.5% DW and a biomass production of 25.1 g FW in flask containing 100 mL of medium. In our hands, increasing sucrose concentration beyond 6% (*w*/*v*) did not resulted in higher ATL accumulation but have a detrimental impact on growth (data not shown). Baldi et al. [42] observed that, compared to glucose, the use of sucrose as carbon source resulted in both higher biomass and PPT accumulation in *L. album* cell suspension. Similarly Kadkade [44] have observed a better PPT production in *Podophyllum peltatum* callus using sucrose than with maltose. In the same way, Schmitt and Petersen [45] observed both pinoresinol and matairesinol accumulation increase in *Forsythia intermedia* cell suspension adding 6% (*w*/*v*) compared to 2% (*w*/*v*) sucrose. Van Uden et al. [46] also used 6% (*w*/*v*) sucrose to obtain optimal biomass and 1% DW MPT accumulation in *L. flavum* non transgenic root lines. Chattopadhyay et al. [47,48] increased up to 7.2% (*w*/*v*) glucose for optimal production of 4.26 mg/L PPT in *P. hexandrum* cell suspensions producing a biomass of 6.5 g DW per liter of suspension.

### 2.5. Impact of Phytohormones and Elicitors Treatments

**Figure 5:**
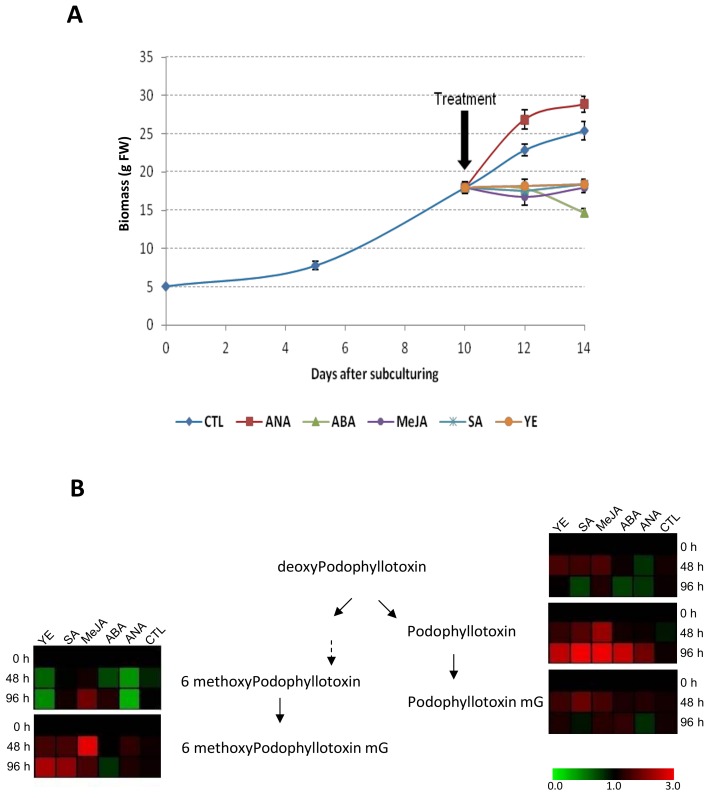
Impact of phytohormones and elicitors treatments on the growth and ATL accumulation profiles of HRLF15.2 line. (**A**) Growth curves of HRLF15.2 line treated with NAA (1 mg/mL), ABA (100 µM), MeJA (100 µM), SA (100 µM) or YE (3% *w*/*v*); (**B**) Relative impact of NAA (1 mg/mL), ABA (100 µM), MeJA (100 µM), SA (100 µM) or YE (3% *w*/*v*) treatments on the intracellular accumulation kinetic of the main ATL and their glucosylated forms in HRLF15.2 line. Detailed ATL accumulation kinetics are presented in Figure S3. Each point is the mean and standard deviation of the three independent experiments.

The impacts of phytohormones and elicitors treatments on biomass and ATL accumulation were evaluated by medium supplementation with phytohormones involved in root growth and development (i.e., the synthetic auxin NAA), in stress response (salicylic acid, SA and methyl jasmonate, MeJA) or both (abscisic acid, ABA) as well as fungal elicitors (yeast extract, YE). The results are reported in Figure 5 and Figure S3.

All treatment resulted in stimulation of ATL secretion into the culture medium except for NAA treatment perhaps because of cell wall reinforcement. Lignin and lignans share and possibly compete the same precursors, and auxin treatment have been previously shown to stimulate lignin biosynthesis at the expense of lignan accumulation in *Pinus taeda* cell cultures [49]. From all the treatments tested here only NAA induced an enhancement of biomass production. However this biomass increase resulted in only a slight increase in ATL, mainly in MPTG; therefore the productivity did not take advantage of this biomass increase.

On the contrary, MeJA stimulated the ATL accumulation since a 2-fold increase in MPTG was observed 48 h post-treatment with a total MPT content of 7.5% DW. Ionkova et al. [50] also reported a slight increase of MPTG (up to 3.6% DW) and its dimethyl derivatives (up to 3.1% DW) accumulation in response to 150 µM MeJA treatment in HR cultures of *Linum tauricum*. In our hands, ATL production increase resulting from this treatment was at the expense of biomass production resulting in an overall decrease of ATL yield. These results evidence the difficulty to obtain productivity gains for specialized metabolite production using such approaches.

SA showed a comparable effect to YE treatment with very similar traits: less biomass accumulation and very specific stimulation in ATL biosynthesis with a differential accumulation of the aglycone vs. glycoside forms of MPT and PPT and having less impact on DPT biosynthesis. This response similarity observed for SA and YE is not surprising as SA is an essential mediator for fungal stress signaling [51]. Interestingly these treatments induced differential stimulation of algycone *vs*. glucoside ATL forms: stimulation of MPTG and PPT accumulation whereas at the same time MPT and PPTG were far less impacted by these treatments. These results showing that the glycosylation step certainly involved substrate specific glucosyltransferases (Figure 1) with possible distinct spatio-temporal regulation of these enzymes. In our hands, using YE treatment, the highest total MPT content of 5.6% DW was measured 96 h post-treatment.

Finally, ABA treatment resulted only in a late (96 h post treatment) and specific stimulation of PPT biosynthesis that could rely on a differential induction of enzymes acting on DPT (DOP7H leading to PPT vs. DOP6H leading to MPT (Figure 1)) and without stimulating the PPT glycosylation step observed with both SA and YE treatments.

Altogether, these results evidenced for the first time a specific induction of distinct branches of the ATL biosynthesis depending on the treatment that could reflect very specific roles for these compounds in planta. This specific induction could also be useful to orientate the metabolic flux to the production of a single ATL.

### 2.6. Precursors Feeding

Precursor feeding strategy using cheap precursors was evaluated using l-Phe, an early biosynthetic precursor at the entry point of the phenylpropanoid pathway, and ferulic acid, a late precursor at the entry point for the biosynthesis of coniferyl alcohol through monolignols pathway. Feeding experiments with young shoots of *F. intermedia* have shown that both l-Phe and ferulic acid are good precursors for lignan biosynthesis [52].

**Figure 6:**
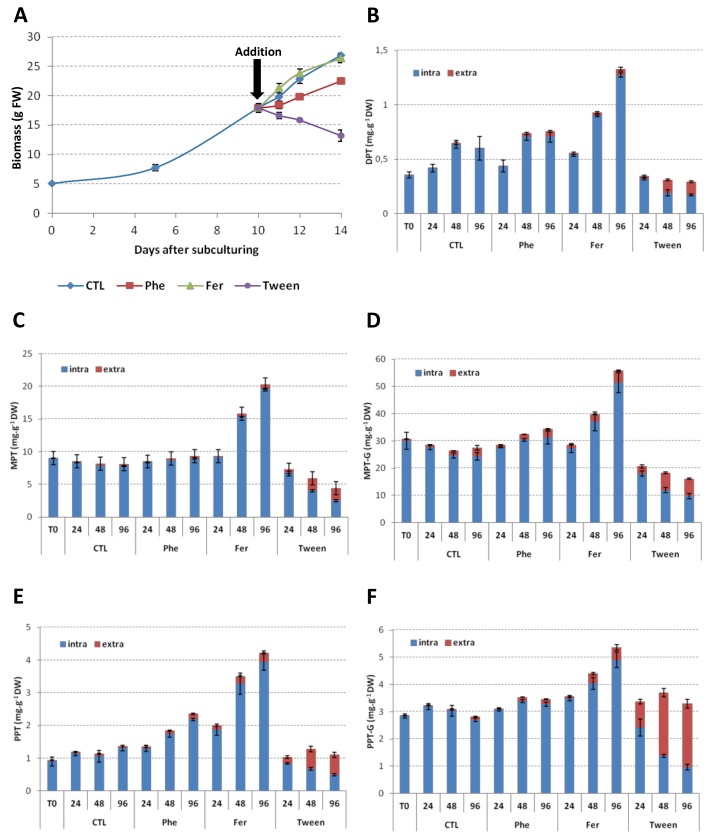
Impact of precursors feeding and permeation on the growth and ATL accumulation profiles of HRLF15.2 line. (**A**) Growth curves of HRLF15.2 line fed with l-Phe (1 mM) or ferulic acid (1 mM) or treated with Tween-20 (2% *w*/*v*); (**B**) Impact of l-Phe (1 mM) or ferulic acid (1 mM) or Tween-20 (2% *w*/*v*) addition on the intracellular and extracellular accumulation kinetic of DPT in HRLF15.2 line; (**C**) Impact of l-Phe (1 mM) or ferulic acid (1 mM) or Tween-20 (2% *w*/*v*) addition on the intracellular and extracellular accumulation kinetic of MPT in HRLF15.2 line; (**D**) Impact of l-Phe (1 mM) or ferulic acid (1 mM) or Tween-20 (2% *w*/*v*) addition on the intracellular and extracellular accumulation kinetic of MPTG in HRLF15.2 line; (**E**) Impact of l-Phe (1 mM) or ferulic acid (1 mM) or Tween-20 (2% *w*/*v*) addition on the intracellular and extracellular accumulation kinetic of PPT in HRLF15.2 line; (**F**) Impact of l-Phe (1 mM) or ferulic acid (1 mM) or Tween-20 (2% *w*/*v*) addition on the intracellular and extracellular accumulation kinetic of PPTG in HRLF15.2 line. Data on l-Phe or ferulic acid uptakes are presented Figure S4. Each point is the mean and standard deviation of the three independent experiments.

Both l-Phe and ferulic acid cellular uptakes were assessed by their disappearance from the culture medium associated with a simultaneous transient cellular accumulation in fed HR followed by a decrease because of their metabolization (Figure S4). l-Phe feeding resulted in a biomass decrease (Figure 6A), reduced viability (data not shown) and, except for PPT, was associated with only a slight increase in ATL accumulation (Figure 6B–F, Table S4). As observed for ABA treatment, a differential stimulation of DOP6H leading to a more important accumulation of PPT at the expense of DOP7H is here suspected. Therefore, both ABA treatment and l-Phe feeding could constitute attractive strategies to isolate the cDNA encoding for both DOP6H and DOP7H using subtractive library strategy associated with high throughput sequencing. The cellular toxicity and stress associated with l-Phe feeding on plant cells have been previously reported [53,54]. l-Phe feeding of *L. flavum* cells has previously resulted in contrasting effects: Van Uden et al. [54] described an increase of MPT accumulation in cell suspension, whereas Berlin et al. [18,19] observed only an increase in the lignan precursor coniferin accumulation in untransformed roots. The fact that l-Phe is a precursor for a wide range of secondary metabolites as well as for the synthesis of protein and other primary metabolites could explain these results.

Ferulic acid is more soluble in culture media than coniferyl alcohol and is a cheaper precursor than early lignan intermediates such as pinoresinol. Moreover, here it did not present a detrimental impact on cellular viability as observed for l-Phe and its addition in HR culture medium resulted in a 2-fold increase in both cellular and secreted ATL accumulation with a total MPT content reaching 7.2% DW 96 h after ferulic acid feeding (Figure 6B–F, Table S4). Considering these results, ferulic acid feeding appeared as an attractive strategy to increase ATL accumulation.

### 2.7. Permeation Experiments

Cell and tissue cultures excreting secondary metabolites into culture medium are preferred for biotechnological approaches because it allows an easier purification process of the desired metabolite. Here Tween-20 was used to permeate HR in order to release ATL into the culture medium. In our hands, permeation with Tween 20 effectively resulted in a release of ATL that start 24 h after its addition and was maximum at 48 h (Figure 6). This permeation also led to a decrease of biomass production and cell viability attributed both to the effect of Tween (for example G6PD activity was detected in extracellular medium after treatment (Figure S5) and probably also because of the toxicity of ATL themselves. Here, ATL glycosides were more likely to be secreted/transported. Future work will be conducted to elucidate this observation.

Despite this negative impact on viability and growth, these results indicate that the release of ATL into the culture medium for easier extraction and purification processes is feasible. In such a context, the use of batch culturing could solve this biomass issue in order to create biomass first, and then proceed to permeation treatment to release ATL. The surviving roots can be subcultured and the cycle starts again. Future research will be conducted on the development and scale-up of such two-phase system, which could facilitate the secretion of ATL from HR, enhance their productions, simplify their extraction and thereby reduce the costs. The use of specific transporters to secrete ATL into the culture medium could also be attractive for future experiments. Indeed, plant roots exude a substantial amount of fixed carbon biomass into the surrounding media/matrix. It is estimated that as much as 20% of the fixed carbon biomass is exuded from roots [55]. Exudation of specialized metabolites by HR has been reviewed by Cai et al. [56]. Root exudates contain polysaccharides and a number of specialized metabolites but there is a lack of knowledge concerning the molecules responsible for root exudation, peculiarly for lignans. The ABC-type transporters play a role [57] but it is probable that other transporter could also contribute to root exudation. Thus, following their identification, the use of specific phytochemical transporters localized at the plasma membrane surface could be future desirable targets for modulating ATL exudation into the culture medium for easier extraction and purification processes.

## 3. Materials and Methods

### 3.1. Plant Materials

Seeds of *Linum flavum* were a gift of Claire Doré from INRA Rennes, France. Plants were harvested at the garden of the “Pôle Universitaire d’Eure et Loir, Chartres” and a voucher specimen was deposited under the number LF-01 in our herbarium.

### 3.2. Agrobacterium Rhizogenes Strain and Culture Condition

*Agrobacterium rhizogenes* strains ATCC 15834 and LBA 9402 were used. These strains were subcultured in YEB medium consisting of 0.5% (*w*/*v*) beef extract, 0.5% (*w*/*v*) polypeptone, 0.5% (*w*/*v*) sucrose, 0.1% (*w*/*v*) yeast extract, and 0.05% (*w*/*v*) MgSO_4_ pH7.0.

### 3.3. Chemicals

All chemical for plant treatments were purchased from Sigma-Aldrich (Saint-Quentin Fallavier, France). Reagents for extraction, purification and HPLC analysis were of analytical grade or higher available purity (Thermo Fischer Scientific, Villebon-sur-Yvette, France). Water was produced by a Milli-Q water-purification system (Millipore, Fontenay sous Bois, France). Solutions were filtered through 0.45 µm nylon membranes prior to use for HPLC. The *o*-coumaric acid (internal standard) was purchased from Sigma-Aldrich and the PPT standard from Chromadex (Irvine, CA, USA). DPT was purified from *Anthriscus sylvestris* as previously described by Van Uden et al. [58]. MPT and MPT-G were purified from *Linum flavum* roots as described by Doussot et al. [59].

### 3.4. Plant Transformation

The seeds were soaked briefly in 70% (*v*/*v*) ethanol, then surface sterilized by soaking in 2.5% (*v*/*v*) sodium hypochlorite solution for 15 min, and finally rinsed three times with sterile double distilled water. The seeds were germinated on hormone-free MS medium (Meridis, Montpellier, France) containing 0.28% (*w*/*v*) Phytagar, and seedlings were maintained aseptically on MS medium supplemented with 3% (*w*/*v*) sucrose at 25 °C under a 16 h/8 h light/dark cycle (30 μmol/m^2^·s photosynthetically active radiations).

Hypocotyls of about 0.5 cm and cotyledon discs of about 1 cm^2^ of aseptically grown *L. flavum* plantlets were immersed in a bacterial suspension (overnight grown in YEB liquid medium up to A_650nm_ = 1) for 2 h; then blotted dry on sterile filter-paper to remove excess bacteria and placed on culture plates. In addition, control explants were immersed in sterile distilled water and incubated in the same conditions as control. After 2 days of co-culture at 25 °C in the dark, the explants were transferred onto a MS hormone-free media containing 500 mg·L^−1^ Timentin (15:1 (*w*/*w*) mixture of ticarcillin disodium and potassium clavulanate (Meridis)) to eliminate bacteria and then incubated at 25 °C in the dark. Transformed roots appear within 14 ± 2 days after infection. Each hairy root line was isolated, identified and subcultured every 14 days in hormone-free MS medium containing Timentin (500 mg·L^−1^) and incubated on a rotary shaker at 100 rpm at 25 °C in the dark.

### 3.5. DNA Extraction and PCR Analysis

Genomic DNA was extracted from bacterium-free *L. flavum* hairy roots and natural (untransformed) roots using CTAB method as described by Doyle and Doyle [60].

Polymerase chain reaction (PCR) identification of the rooting locus genes *ROL-B* and *ROL-C* was performed using DNA from the hairy roots as template and the non-transformed roots as control, respectively. The following primers were used for amplification of *ROL-B* and *ROL-C* sequences: ROLB-R (5′-GCTCTTGCAGTGCTAGATTT-3′) and ROLB-F (5′-GAAGGTGCAAGCTACCTCTC-3′), ROLC-F (5′-CTCCTGACATCAAACTCGTC-3′) and ROLC-R (5′-TGCTTCGAGTTATGGGTACA-3′), respectively. The V*ir*D2 bacterial gene, not transferred to the plant genome during transformation, was used as control to verify the absence of agrobacteria in the transformed lines. The specific primers for the detection of V*IR-* D2 used were: VIRD2-F (5′-ATGCCCGATCGAGCTCAAGT-3′) and VIRD2-R (5′-CCTGACCCAAACATCTCG-GCTGCCCA-3′).

For amplification, the PCR parameters consisted of a step of 4 min at 94 °C and 35 cycles (each consisting of 1 min at 94 °C, 1 min at 55 °C and 1 min at 72 °C), followed by a final extension at 72 °C for 10 min.

The PCR products were analyzed by electrophoresis on a 1.0% agarose gel using Tris-Acetate-EDTA buffer and the bands visualized under ultraviolet light at a wavelength of 260 nm following their staining with Ethidium Bromide.

### 3.6. RNA Extraction and Semi-Quantitative RT-PCR Analysis

Total RNA were extracted using the RNeasy Plant Mini kit (Qiagen, Hilden, Germany) and submitted to a treatment with RNase free DNase (Promega, Charbonnières-les-Bains, France). RNA were quantified using a fluorometer and the Quant-iT RNA Assay Kit adapted for the Qubit fluorometer according to the manufacturer’s protocol (Thermo Fisher Scientific).

First-strand cDNA was synthesised at 37 °C for 1 h. Briefly, 0.5 µg of total RNA was incubated in reverse transcription buffer with 4 units of Omniscript Reverse Transcriptase (Qiagen), 0.5 mM of each dNTP, 1 µM of oligo-dT primer and 10 units of RNAse inhibitor (RNasin, Promega). A 540 bp fragment of the *ROL-B* cDNA was amplified using the specific primers ROLB-R (5′-GCTCTTGCAGTGCTAGATTT-3′) and ROLB-F (5′-GAAGGTGCAAGCTACCTCTC-3′). A 770 bp fragment of the *ROL-C* cDNA was amplified using the specific primers ROLC-F (5′-CTCCTGACATCAAACTCGTC-3′) and ROLC-R (5′-TGCTTCGAGTTATGGGTACA-3′). To normalize the amount of mRNA in each PCR reaction, a PCR product corresponding to the second exon of the *ACTIN-2* gene was amplified with ACT-F2 forward primer (5′-TCTGGAGATGGTGTGAGCCACAC-3′) and ACT-R2 reverse primer (5′-GGAAGGTACTGAGGGAGGCCAAG-3′) designed from the tobacco sequence. cDNA fragments were amplified during 23 cycles.

### 3.7. HR Treatments

Following transfer to fresh medium, HR culture was treated at day 10 by (i) different phytohormones: naphthaleneacetic acid (NAA,1 mg/mL final concentration, or abscisic acid (ABA, 100 µM final concentration)); (ii) different elicitors methyl jasmonate (MeJA, 100 µM final concentration) or salicylic acid (SA, 100 µM final concentration) or yeast extract (YE, 3% (*w*/*v*) final concentration); (iii) different precursors: l-Phe (1 mM final concentration) or ferulic acid (1 mM final concentration) or (iv) permeabilisant: Tween-20 (2% (*w*/*v*) final concentration) [61]. At the same time, control HR were inoculated in the same MS medium with sterile double distilled water and used as reference during this study. Incubation continued on a gyratory shaker at 120 rpm in darkness at 25 °C. Treated and control HR were collected for analyses after elicitation.

### 3.8. MPT and MPTG Purification

MPT and MTPG purifications were performed according the method described by Van Uden et al. (1992) [62]. Their identification was confirmed by LC-MS and NMR. The data obtained are in full agreement with literature data for MPT [62] and for MPTG [63]). NMR spectra were acquired at 300 K on a Bruker (Bremen, Germany) Avance III 600 spectrometer (Magnet system 14.09 T 600 MHz/54 mm) operating at 600.17 MHz for 1H, using a TXI 5 mm z-gradients probe. The TOPSPIN (Bruker) software was used. Shim control was performed automatically by gradient shimming and final lineshape optimization (Topshim 1D procedure). ^1^H-NMR spectra were obtained using a classical proton sequence (90° proton pulse was calibrated to 7.36 µs at −1 dB (18.34 W)), 6602 Hz spectral width and 7 s relaxation delay. Each spectrum consisted of 4 dummy scans and 128 scans of 64 K data points. The free induction decay was multiplied by an exponential weighing function corresponding to a line broadening of 0.3 Hz prior to Fourier transformation. The non-zero filled obtained spectra were manually phased and baseline-corrected, calibrated using the residual solvent resonance.

### 3.9. ATLs Extraction and Quantification by UPLC-HR-MS

LC-HR-MS was performed on a Bruker maXis UHR Q-TOFmass spectrometer coupled to an Ultimate 3000 RSLC system (Dionex, Germering, Germany) with a binary pump, an autosampler, a column thermostated at 40 °C, and a DAD detector. A Dionex Acclaim RSLC 120 C18 (250 × 2.1 mm; 2.2 μm) column fitted with a C18 Security Guard Ultra (2.1 mm) (Phenomenex, Le Pecq, France) guard filter was used for LC separations. The mobile phase was water + 0.1% formic acid (solvent A) and acetonitrile + 0.08% formic acid (solvent B). A solvent gradient at a flow rate of 300 μL/min was set as follows: 3% of B for 1 min, then a linear gradient up to 70% of B during 39 min. The column was rinsed with 95% of B for 5 min and then re-equilibrated to the initial conditions for 10 min after each run. 4 μL of sample reconstituted in methanol at 10 mg/mL of engaged material were injected. UV spectra were recorded from 195 to 500 nm. The positive mode using an ESI source in the range of 50 to 1300 *m*/*z* at 1 Hz was used to acquire MS data. The parameters were as follows: capillary voltage 4.5 kV, nebulizing gas at 1.5 bar, drying gas heated at 200 °C, at 8 L/min. Data (MS and UV) were acquired with Compass 1.3 software (Bruker) and MS areas were measured with Quant Analysis 2.1 software (Bruker). Extracted ion chromatograms for DPT, PPT, PPTG, MPT and MPTG are presented in Figures S6–S10.

### 3.10. Standard Curves for Quantification

PPT, MPT and MPT-G were identified by comparison of their retention times and their UV spectra to those of standards and quantitated against 5-point calibration curves for PPT, MPT and MPT-G (Tables S1 and S2), with *o*-coumaric acid as the internal standard.

### 3.11. Statistical Analysis

All data presented are the means and standard deviation of at least three independent replicates. When applicable, the one-way analysis of variance (ANOVA) was determined using XL-stat2016 software (Addinsoft, Paris, France) and the sample means were distinguished using the Student Newman-Keuls method (*p* < 0.05).

## 4. Conclusions

To conclude, this work presents new results on ATL production from HR lines of a wild flax species *L. flavum*. Here we report the establishment of *L. flavum* HR lines as well as their physiological, molecular and biochemical characterizations leading to the selection of a highly productive HR line. Following selection, the influence of culture medium, carbon source, hormone, elicitation, precursor feeding and permeation on ATL production have evidenced *L. flavum* HR cultures as a scalable platform for the production of anticancer ATL. Here we show that following the choice of the right and stable genotype, low nitrogen containing medium, high sugar content, MeJa treatment as well as ferulic acid feeding could result in high production rate of total ATL from HR of *L. flavum*: up to 9% (*w*/*w*) production of total ATL on DW basis corresponding to a potential bioproductivity of *ca* 4.5 g/L. Our results also evidence the usefulness of permeation to simplify and decrease the cost of purification process. Altogether, these prospective results clearly demonstrate that *L. flavum* HR constitutes a promising experimental system for the elucidation of ATL biosynthetic pathway and its regulation but also as a promising biotechnological production system. This could be of special interest for future biotechnological approaches.

## Figures and Tables

**Table 1:** Lignans profiling and growth index of wild type in vitro or transgenic hairy roots of *L. flavum* cultivated on hormone free B5-derived medium.

Cell Lines	MPT-G mg·g^−1^ DW	MPT mg·g^−1^ DW	PPT-G mg·g^−1^ DW	PPT mg·g^−1^ DW	DPT mg·g^−1^ DW	Growth Index Days 0–20
in vitro WT Lf root	4.9 ± 0.5	1.7 ± 0.1	1.0 ± 0.3	0.1 ± 0.1	0.2 ± 0.1	1.3 ± 0.1
HRLF15.1	21.9 ± 4.4	8.3 ± 1.6	2.3 ± 0.4	0.6 ± 0.1	1.3 ± 0.4	3.6 ± 0.1
HRLF15.2	22.6 ± 5.7	7.5 ± 1.0	2.9 ± 0.8	0.8 ± 0.3	1.0 ± 0.2	4.0 ± 0.2
HRLF15.3	5.8 ± 0.1	5.1 ± 0.6	1.6 ± 0.8	0.3 ± 0.1	0.6 ± 0.1	3.5 ± 0.1
HRLF15.4	6.1 ± 0.2	3.0 ± 0.3	1.3 ± 0.3	0.2 ± 0.1	0.9 ± 0.1	3.4 ± 0.1
HRLF15.5	5.8 ± 1.3	1.5 ± 0.1	2.7 ± 0.5	0.4 ± 0.2	0.5 ± 0.3	3.8 ± 0.1
HRLF94.1	9.7 ± 0.6	3.5 ± 0.2	1.2 ± 0.3	0.2 ± 0.1	1.0 ± 0.4	5.0 ± 0.2
HRLF94.2	24.1 ± 3.1	8.4 ± 0.4	4.9 ± 0.3	0.6 ± 0.2	1.6 ± 0.4	5.9 ± 0.2
HRLF94.3	5.9 ± 0.2	5.6 ± 1.2	2.0 ± 0.6	0.3 ± 0.1	0.8 ± 0.2	5.2 ± 0.3
HRLF94.4	4.9 ± 1.4	6..3 ± 0.7	1.5 ± 0.4	0.2 ± 0.1	0.7 ± 0.2	5.0 ± 0.2
HRLF94.7	20.4 ± 1.3	3.2 ± 1.2	2.0 ± 0.5	0.3 ± 0.1	0.8 ± 0.2	4.4 ± 0.2
HRLF94.8	4.1 ± 0.3	6.9 ± 1.1	1.2 ± 0.3	0.2 ± 0.1	0.7 ± 0.2	4.9 ± 0.2
HRLF94.9	12.4 ± 1.9	6.3 ± 2.0	1.8 ± 0.2	0.2 ± 0.1	0.6 ± 0.2	4.8 ± 0.2

Values are the means (±SD) of four replicates.

## Supplementary Materials

Supplementary materials can be found at http://www.mdpi.com/1422-0067/19/4/990/s1.
