# Supplementary material for: Investigation of Linum flavum (L.) Hairy Root Cultures for the Production of Anticancer Aryltetralin Lignans
Source: Int J Mol Sci. 2018 Mar 26;19(4):990. doi: 10.3390/ijms19040990 (PMC5979590; doi:10.3390/ijms19040990)
Supplement: Supplementary file 1 [file ijms-19-00990-s001.pdf]

Supplementary Materials: Investigation of Linum flavum (L.) Hairy Root Cultures for the Production of Anticancer Aryltetralin Lignans

Sullivan Renouard, Cyrielle Corbin, Samantha Drouet, Barbara Medvedec, Joël Doussot, Cyril Colas, Benoit Maunit, Avninder S. Bhambra, Eric Gontier, Nathalie Jullian, François Mesnard, Michèle Boitel, Bilal Haider Abbasi, Randolph R. J. Arroo, Eric Lainé, Christophe Hano

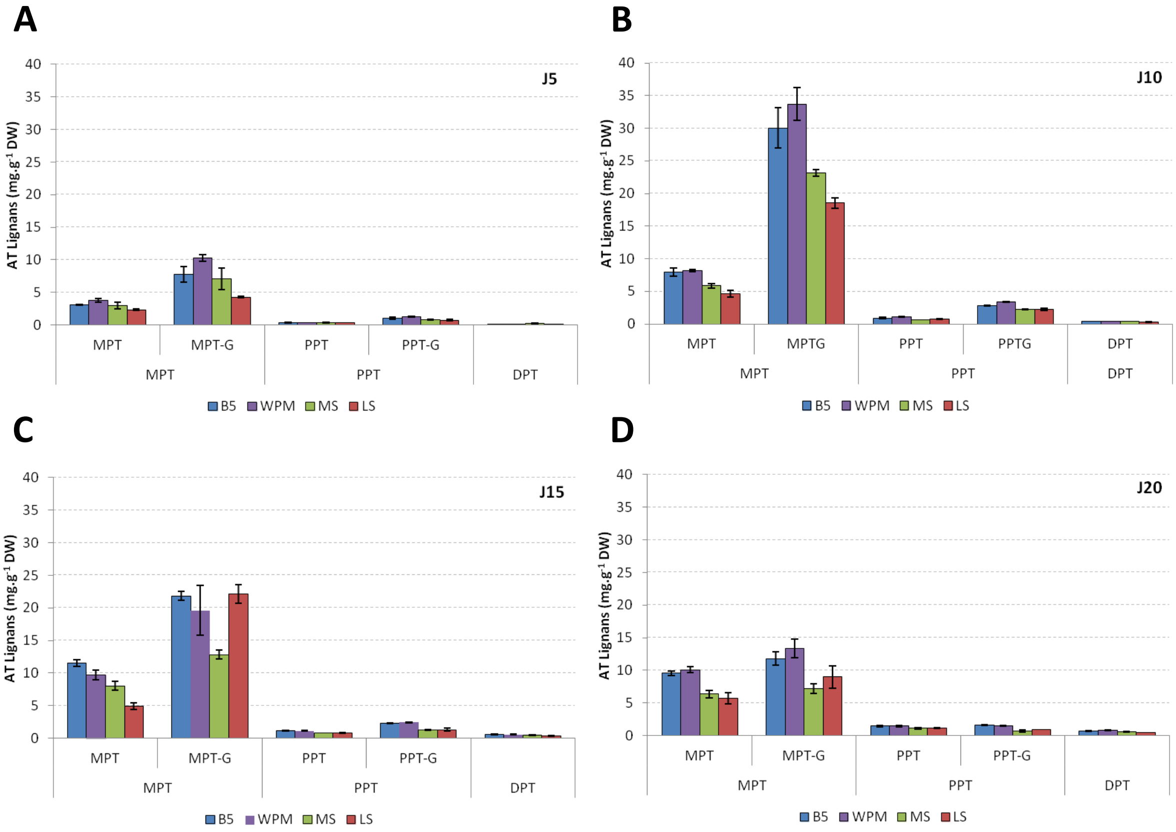

Figure S1. Detailed intracellular ATL accumulation kinetics of the main ATL and their glucosylated forms in HRLF15.2 line growing in the B5, WPM, MS and LS culture media. Each point is the mean and standard deviation of the three independent experiments.

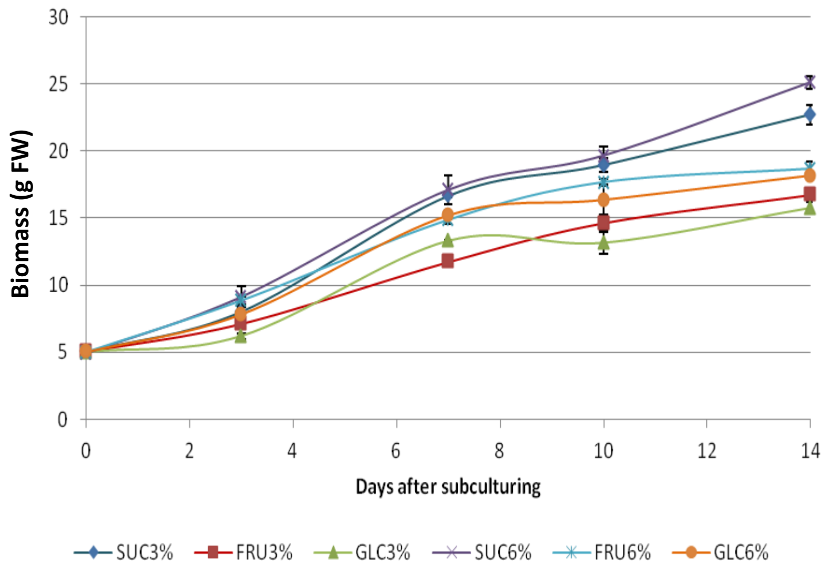

Figure S2. Growth kinetic of the HRLF15.2 line growing in the B5 medium supplemented with 3 or 6% (w/v) sucrose (SUC), fructose (FRU) or glucose (GLC). Each point is the mean and standard deviation of the three independent experiments.

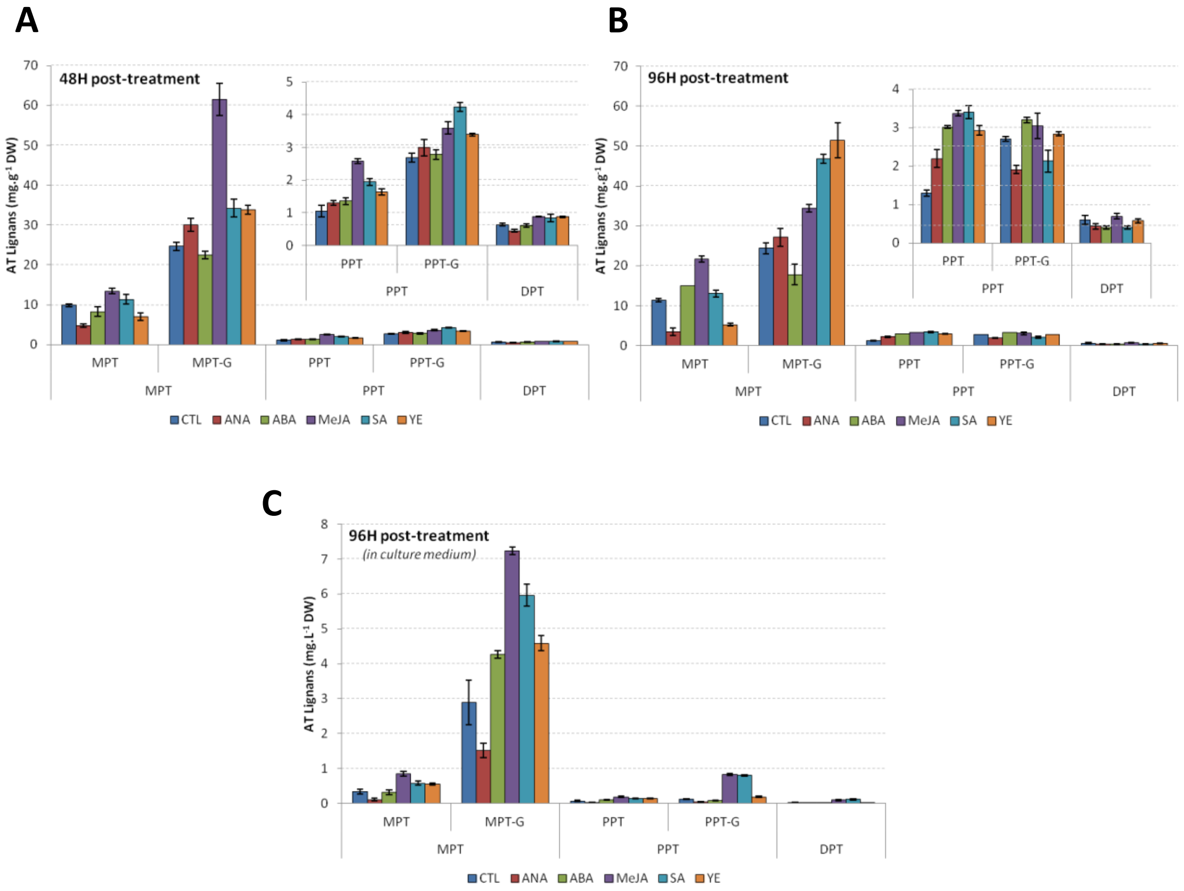

Figure S3. Detailed intracellular ATL accumulation kinetics of the main ATL and their glucosylated forms in HRLF15.2 line treated with ANA (1 mg/ml), ABA (100 µM), MeJA (100 µM), SA (100 µM) or YE (3% w/v). **A.** Intracellular contents measured 48h post treatment; B. Intracellular contents measured 96h post treatment; **C.** Extracellular contents measured 96h post treatment. Each point is the mean and standard deviation of the three independent experiments.

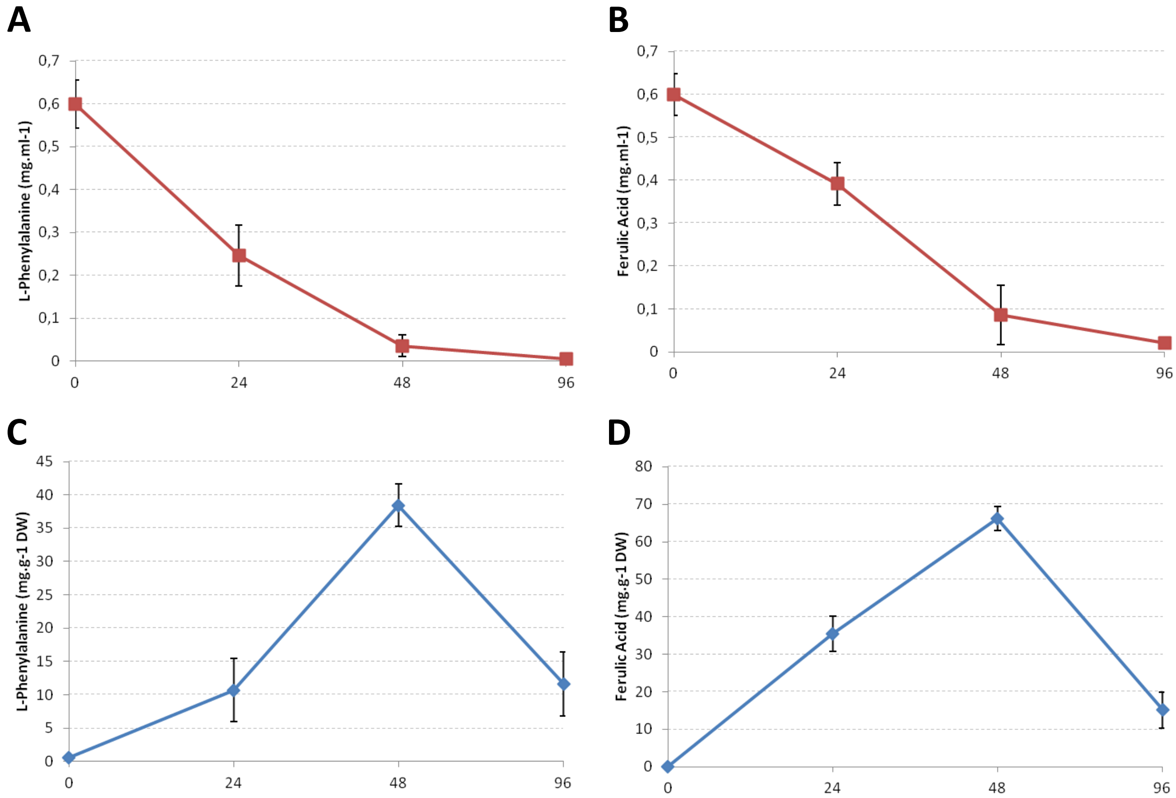

Figure S4. l-Phe or ferulic acid uptakes during precursors feeding of HRLF15.2 line. A. Time course of extracellular l-Phe concentration. B. Time course of extracellular ferulic acid concentration. C. Time course of intracellular l-Phe concentration. D. Time course of intracellular ferulic acid concentration. Each point is the mean and standard deviation of the three independent experiments.

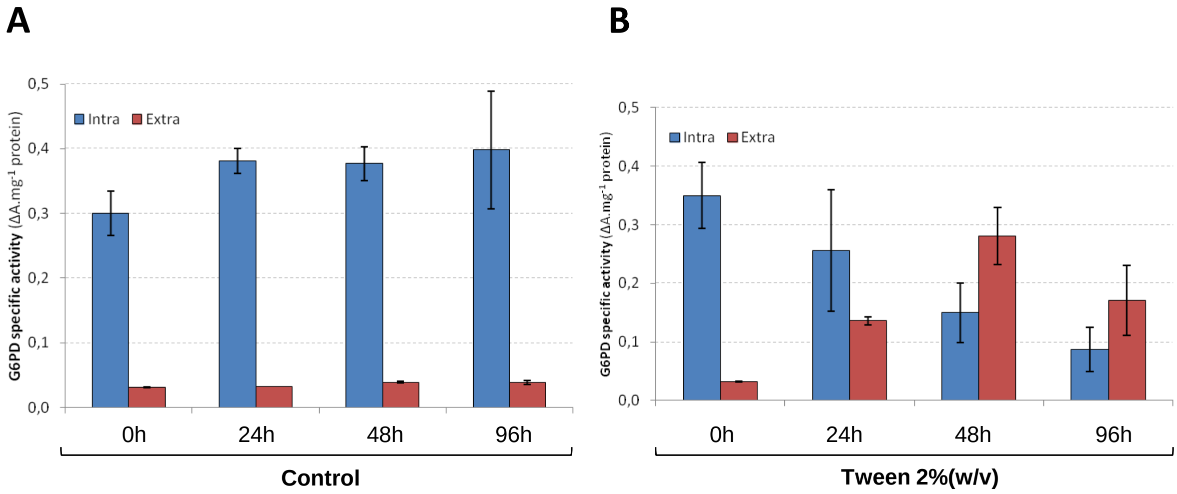

Figure S5. Time course of intracellular and extracellular G6PD activity in control (A) and 2% (w/v) Tween 20-treated (B) HRLF15.2 line. Each point is the mean and standard deviation of the three independent experiments.

DPT

Analysis: X003673CYC.d

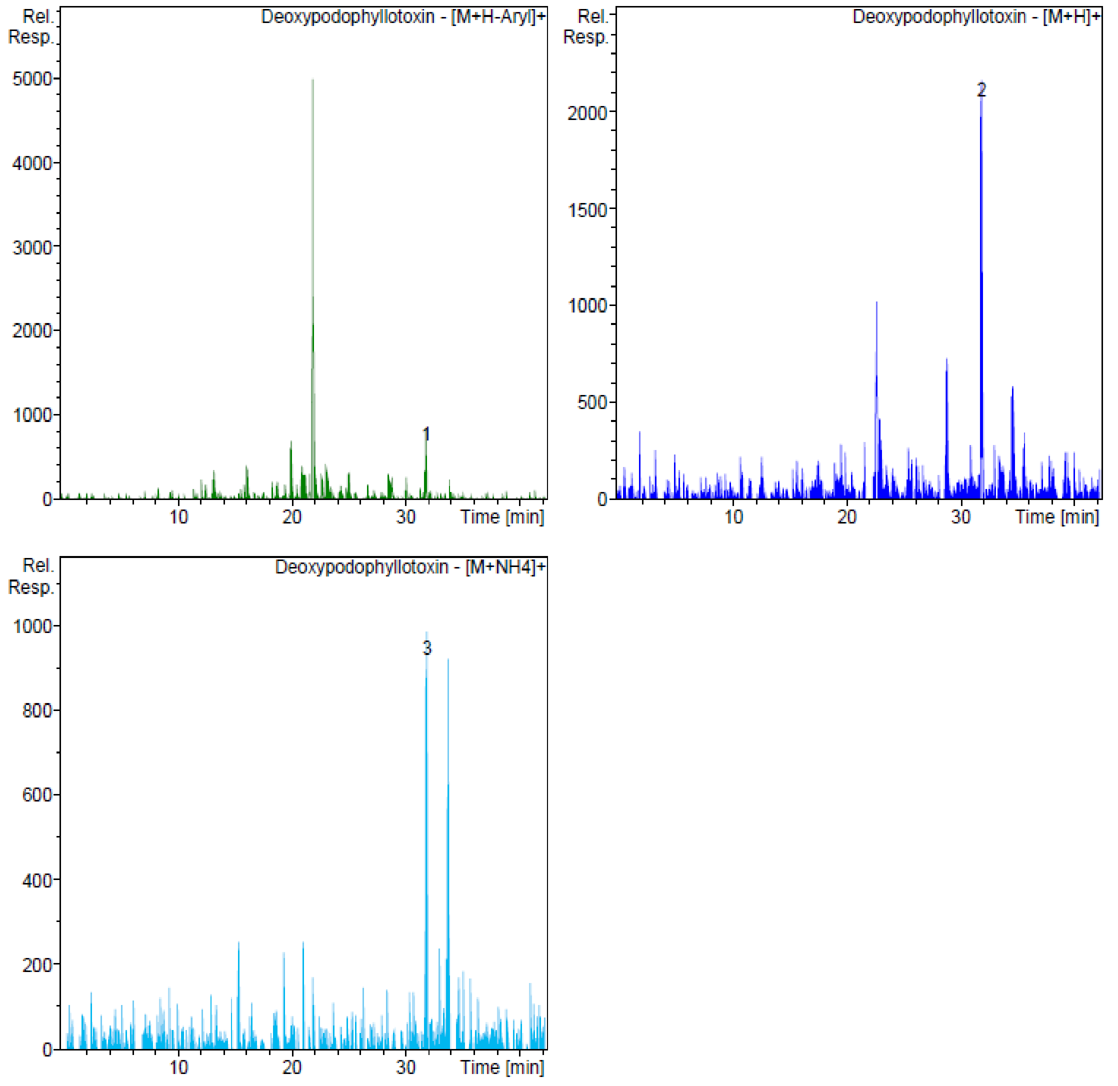

#	Ret.Time	Name	Area	Calc. Conc.	Accuracy
1	31.7	Deoxypodophyllotoxin - [M+H-Aryl]+	4030	n/c	
2	31.7	Deoxypodophyllotoxin - [M+H]+	13556	n/c	
3	31.8	Deoxypodophyllotoxin - [M+NH4]+	4774	n/c	

Figure S6. Extracted Ion Chromatogram for DPT from HRLF15.2 line.

PPT

Analysis: X003673CYC.d

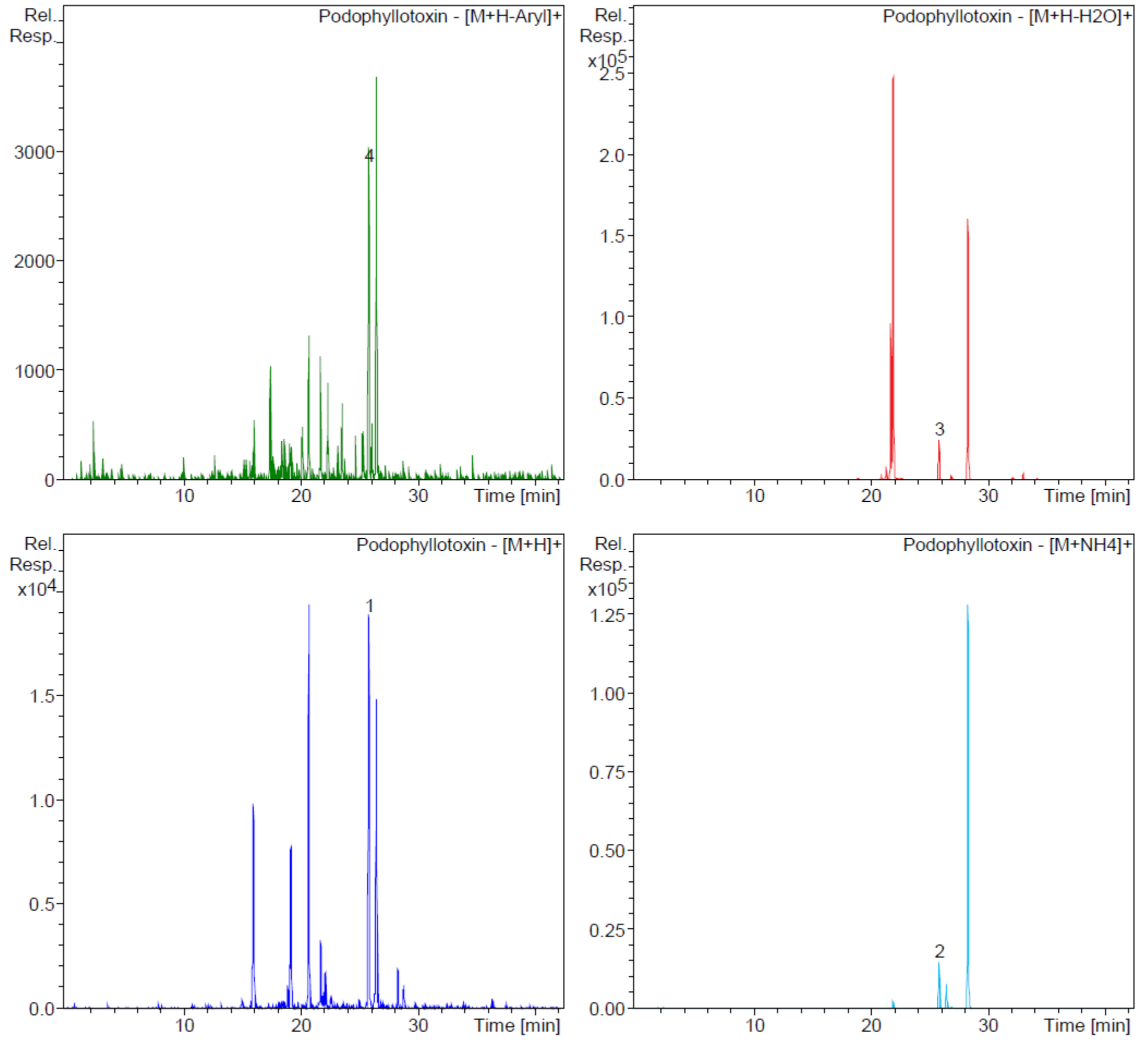

#	Ret.Time	Name	Area	Calc. Conc.	Accuracy
1	25.8	Podophyllotoxin - [M+H]+	121297	n/c	
2	25.8	Podophyllotoxin - [M+NH4]+	87367	n/c	
3	25.8	Podophyllotoxin - [M+H-H2O]+	157837	n/c	
4	25.9	Podophyllotoxin - [M+H-Aryl]+	18537	n/c	

Figure S7. Extracted Ion Chromatogram for PPT from HRLF15.2 line.

PPTG

Analysis: X003673CYC.d

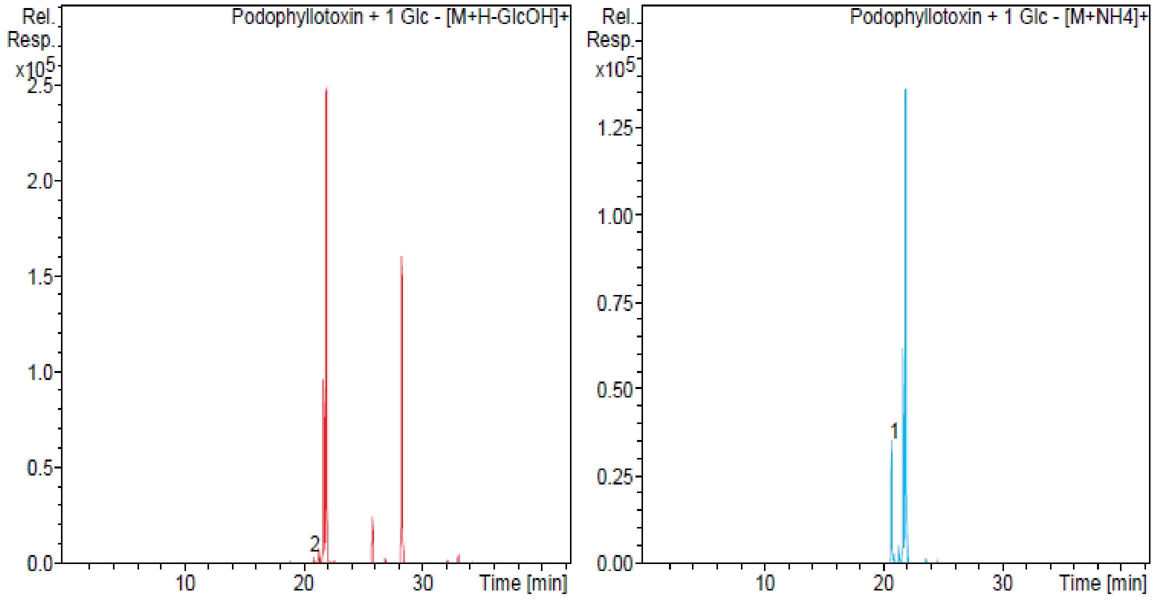

#	Ret.Time	Name	Area	Calc. Conc.	Accuracy
1	21.0	Podophyllotoxin + 1 Glc - [M+NH4]+	16627	n/c	
2	21.0	Podophyllotoxin + 1 Glc - [M+H-GlcOH]+	16518	n/c	

Figure S8. Extracted Ion Chromatogram for PPTG from HRLF15.2 line.

MPT

Analysis: X003673CYC.d

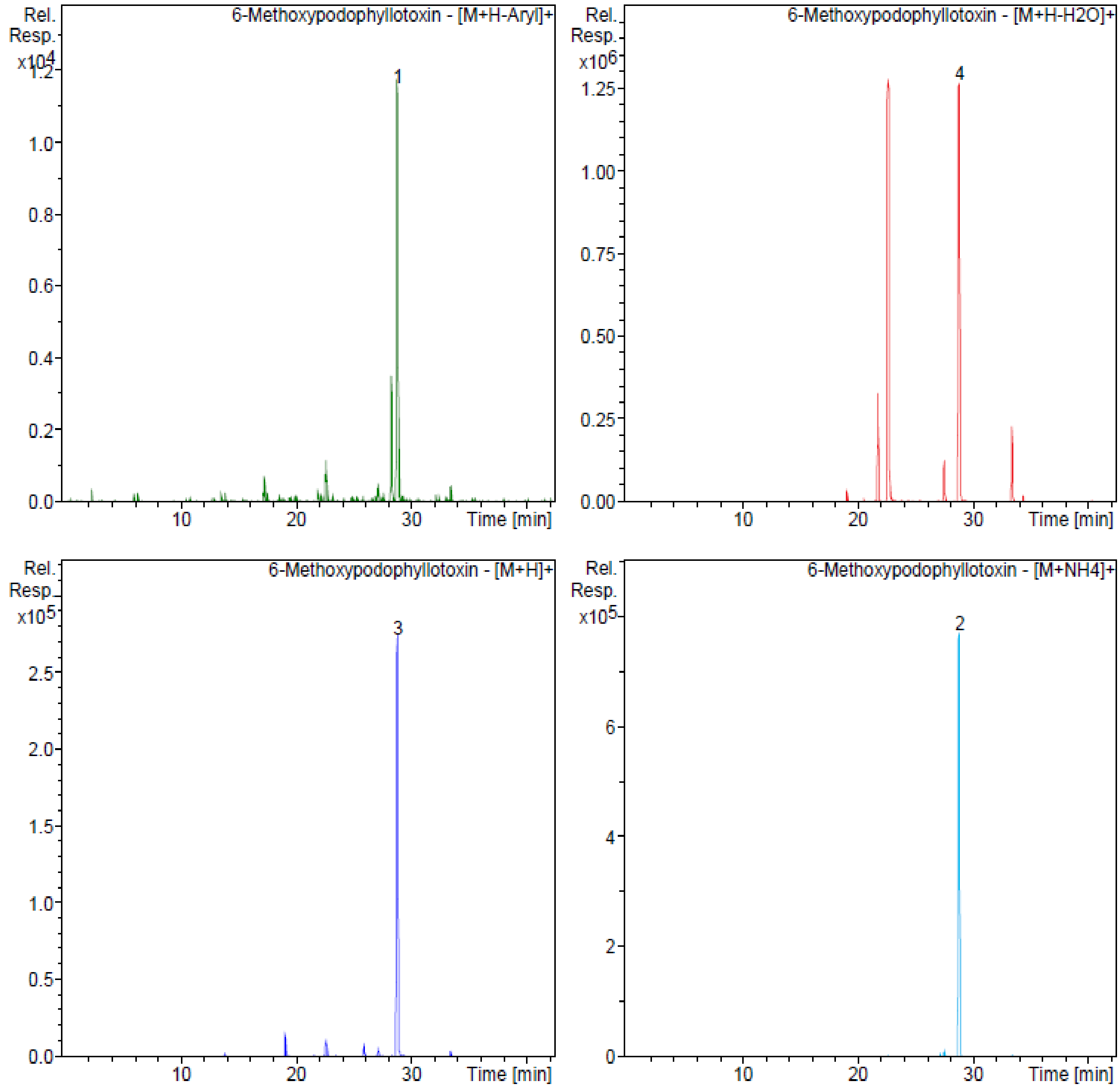

#	Ret.Time	Name	Area	Calc. Conc.	Accuracy
1	28.8	6-Methoxypodophyllotoxin - [M+H-Aryl]+	86265	n/c	
2	28.8	6-Methoxypodophyllotoxin - [M+NH4]+	6198990	n/c	
3	28.8	6-Methoxypodophyllotoxin - [M+H]+	2223010	n/c	
4	28.8	6-Methoxypodophyllotoxin - [M+H-H2O]+	12375094	n/c	

Figure S9. Extracted Ion Chromatogram for MPT from HRLF15.2 line.

MPTG

Analysis: X003673CYC.d

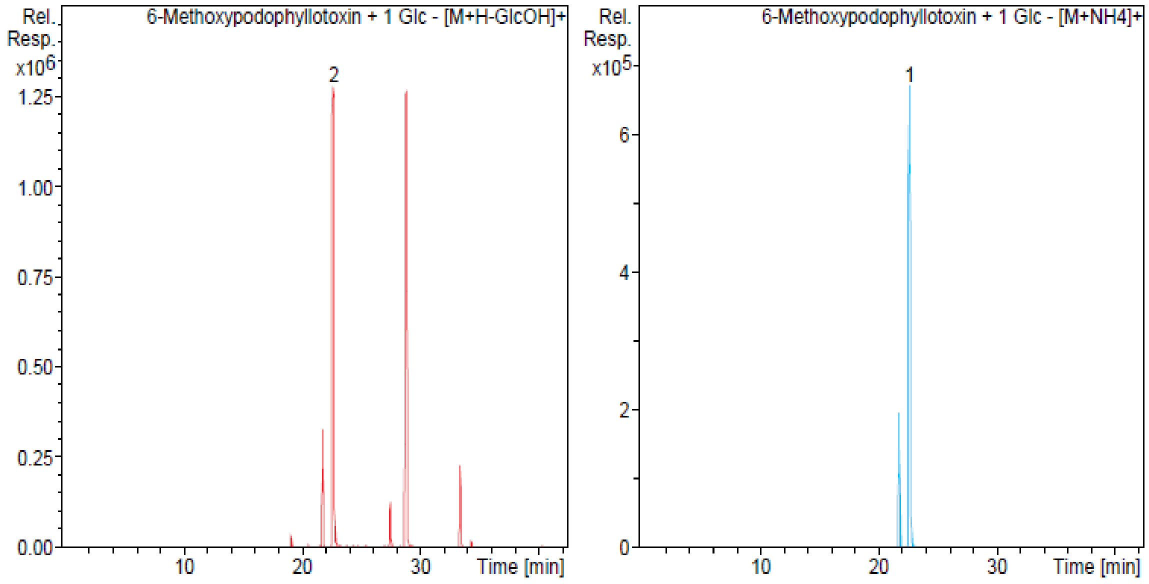

#	Ret.Time	Name	Area	Calc. Conc.	Accuracy
1	22.7	6-Methoxypodophyllotoxin + 1 Glc - [M+NH4]+	5815461	n/c	
2	22.7	6-Methoxypodophyllotoxin + 1 Glc - [M+H-GlcOH]+	13249186	n/c	

Figure S10. Extracted Ion Chromatogram for MPTG from HRLF15.2 line.

Table S1. Lignans identification in *L. flavum* HR cell lines with LC-HR-MS fragmentation.

RT (min)	Compound	Abbreviation	Formula	MW	$[M+Na]^+$	$[M+H]^+$	$[M+NH4]^+$
21.77	podophyllotoxin-7-glucose	PPT-G	C22H32O13	576	599.1735	577.1916	594.2181
22.67	6-methoxypodophyllotoxin-7-glucose	MPT-G	C29H34O14	606	629.1841	607.2021	629.1841
25.93	podophyllotoxin	PPT	C22H22O8	414	437.1207	415.1387	432.1653
28.8	6-methoxypodophyllotoxin	MPT	C23H24O9	444	467.1313	445.1493	462.1759
31.85	deoxypodophyllotoxin	DPT	C22H22O7	398	421.1302	399.1483	416.1748

Table S2. Analytical performance of the proposed method.

Analytes	Calibration	R^2	Range tested µg/mL	LOD µg/mL	LOQ µg/mL
DPT	y = 0.0055x − 0.0024	0.9993	125 - 1000	0.06	0.20
PPT	y=0.0059x – 0.0017	0.9992	125 - 1000	0.09	0.96
MPT-G	y=0.0059x – 0.0116	0.9993	62.5-1000	0.81	2.43
MPT	y=0.0066x – 0.0243	0.9998	62.5-1000	0.37	1.24

Table S3. Ions content of the basal medium, in mM

	Total N	NH_4^+/Total N	PO_4^{2-}	SO_4^{2-}	Ca^{2+}	K^+	Mg^{2+}
MS	60	0.34	1.9	1.5	3	20	1.5
LS	60	0.34	1.9	1.5	3	20	1.5
B5	27	0.08	1.1	2.2	1.1	25	1
WPM	14.7	0.33	1.9	7	3	7	1.5

Table S4. Net synthesis of AT Lignans during supplementation or permeabilization experiments.

		**Φ (0h-24h)** $mg.g^{-1}.h^{-1}$	**Φ (24h-48h)** $mg.g^{-1}.h^{-1}$	**Φ (48h-96h)** $mg.g^{-1}.h^{-1}$
**MPT-G**	**CTL**	-0.088 ± 0.035	-0.181 ± 0.035	- 0.067 ± 0.010
	**L-Phe**	-0.095 ± 0.015	+0.073 ± 0.020	+0.075 ± 0.053
	**Fer**	-0.091 ± 0.073	+0.378 ± 0.135	+0.523 ± 0.070
	**Tween**	-0.418 ± 0.011	-0.517 ± 0.027	+0.130 ± 0.053
**MPT**	**CTL**	-0.022 ± 0.015	-0.035 ± 0.014	-0.019 ± 0.007
	**L-Phe**	-0.023 ± 0.008	-0.002 ± 0.008	+0.006 ± 0.007
	**Fer**	+0.012 ± 0.004	+0.282 ± 0.015	+0.234 ± 0.022
	**Tween**	-0.073 ± 0.013	-0.130 ± 0.014	-0.063 ± 0.006
**PPT-G**	**CTL**	+0.015 ± 0.005	+0.010 ± 0.007	-0.001 ± 0.001
	**L-Phe**	+0.010 ± 0.002	+0.028 ± 0.002	+0.013 ± 0.002
	**Fer**	+0.029 ± 0.005	+0.064 ± 0.007	+0.052 ± 0.004
	**Tween**	+0.021 ± 0.009	+0.035 ± 0.009	+0.040 ± 0.006
**PPT**	**CTL**	+0.011 ± 0.003	+0.008 ± 0.007	+0.009 ± 0.002
	**L-Phe**	+0.017 ± 0.003	+0.037 ± 0.003	+0.029 ± 0.000
	**Fer**	+0.044 ± 0.009	+0.107 ± 0.013	+0.069 ± 0.007
	**Tween**	+0.004 ± 0.001	+0.014 ± 0.002	+0.009 ± 0.001
**DPT**	**CTL**	+0.003 ± 0.002	+0.012 ± 0.002	+0.005 ± 0.002
	**L-Phe**	+0.003 ± 0.002	+0.016 ± 0.002	+0.008 ± 0.001
	**Fer**	+0.008 ± 0.001	+0.024 ± 0.000	+0.020 ± 0.001
	**Tween**	+0.000 ± 0.001	-0.002 ± 0.002	-0.001 ± 0.000

Values are the means (± SD) of 3 replicates.
